# Integrated health, social, and economic impacts of extreme events: evidence, methods, and tools

**DOI:** 10.3402/gha.v5i0.19837

**Published:** 2012-12-17

**Authors:** Michael Marx, Revati Phalkey, Debarati Guha-Sapir

**Affiliations:** 1 Institute of Public Health, Medical School, University of Heidelberg, Heidelberg, Germany; 2 Centre for Research on the Epidemiology of Disasters (CRED), Université Catholique de Louvain, School of Public Health, Brussels, Belgium

Disasters are rarely natural, hazards always are! Extreme weather events are natural physical phenomena, but it is their interactions with vulnerabilities of human life that makes them disasters. Although definitions vary, disasters are a combination of complex, interdependent, mutually influential factors, in action simultaneously. They are best described as ‘serious disruptions of the functioning of a community causing widespread human, material, social, economic or environmental losses which exceed the ability of the affected community or society to cope using its own resources’ (1).

With this view as a point of departure, this special volume on health and health system impact of natural disasters presents studies culminating from the research carried out under the European Commission's 6th Framework Program ‘Project MICRODIS-Integrated Health, Social and Economic Impacts of Extreme Events: Evidence, Methods and Tools’. It was a joint research project coordinated by the Centre of Research on the Epidemiology of Disasters (CRED) bringing together 19 research institutions and partners from 13 countries across Europe and Asia.

## Why investigate?

The need to investigate natural disasters at micro-levels is pertinent for several reasons. First, in the course of climate change and global warming, there is an observed shift in the frequency of extreme weather event occurrences. Although the causality is widely debated, and the correlations remain un-quantified, the co-occurrence is undeniable. Second, unequal geographic distribution of the disasters and their varying intensities pose special challenges. Higher frequencies of natural disasters occur in low- and middle-income countries, where the population in general is vulnerable to one or the other type of disaster and predominantly the poor are affected (2). Asia represents about three-fifths of the world population and has been worst hit by natural disasters in the past few decades (3). In the year 2011, Asia accounted for 44% of global natural disasters, 86% of global disaster victims, and 75% of global disaster damages. Similar trends were recorded from 2001 to 2010 (4).

**Fig. 1:**
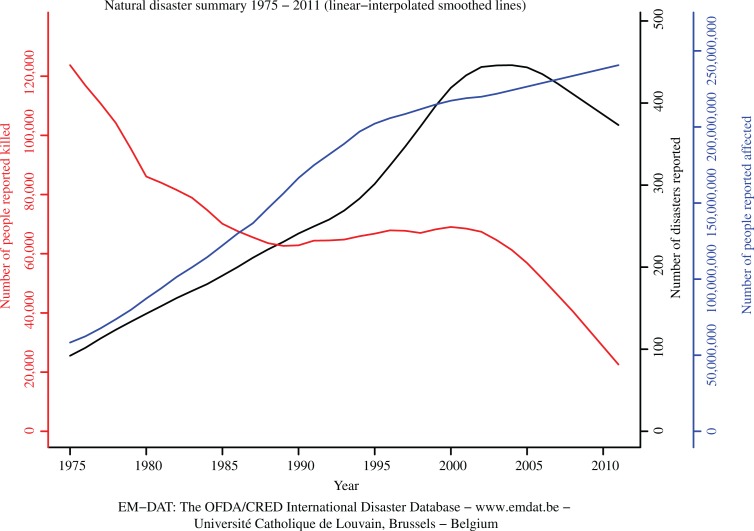
Summary of natural disaster occurrence and human impacts in terms of affected and killed.

Third, specific disasters affect specific geographic areas. In both Asia and Europe, floods, earthquakes, and windstorms accounted for nearly 75% of all extreme events in the past few decades (5). Hydrological disasters accounted for 57% of total disaster victims in 2011. Fourth, the estimated economic losses from natural disasters are rising in an unprecedented way. They were the highest ever registered (235% increase) in 2011 as compared to the annual average damages (US$ 109.3 billion) from 2001 to 2010 (4). Figure 1 illustrates the number of natural disasters reported in the EM-DAT database since 1975–2011 and the number of people affected and killed.

## Health is an important aspect but not the only one!

The World Health Organization (WHO) defines health as ‘a state of complete physical, mental and social well-being and not merely the absence of disease or infirmity’. Health has been a major driver in disaster research and the impacts mainly include mortality, morbidity (injury, infectious diseases, chronic illnesses, and mental health), and disability. Although the frequencies of the different health conditions differ from one disaster type to the other and according to the phases of the disaster, the patterns are similar and few commonalities are identified (6). The number of deaths is considered a reliable indicator of human loss in a disaster. United Nations Development Programme (UNDP) estimates that for every 3,000 people exposed to a disaster, one death is reported clearly indicating that mortality represents only a small part of the impact spectrum (7). Injuries constitute a major part of the morbidity burden following both earthquakes and floods. The injury to mortality ratio is pegged at 3 to 3.5:1, making it a significant health impact concern (8).

It is essential to understand the additional burden produced by natural disasters on already stressed systems. Assessing the impact of disasters is also important to better understand the causalities to better conceive preparedness and coping strategies. Disaster impacts can be placed into three basic categories: health, social, and economic.

**Fig. 2:**
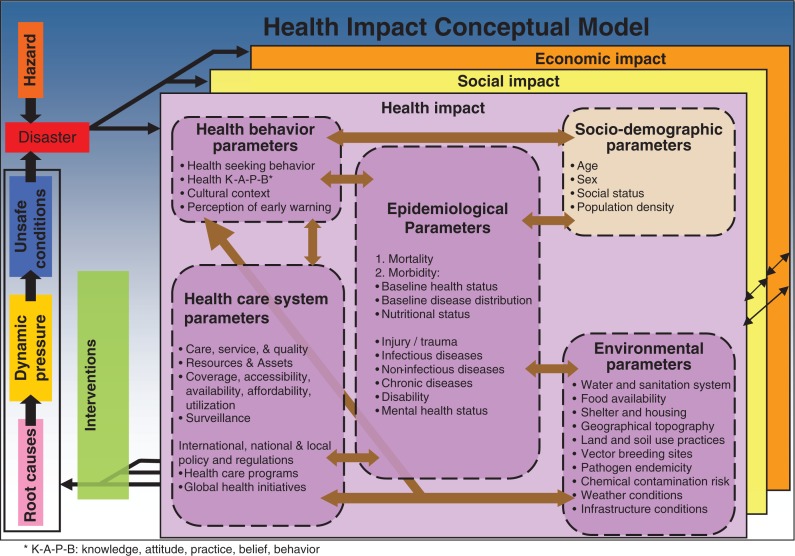
Health and health systems’ impacts of natural disasters.

Health outcomes, even when determined by the individual vulnerabilities, are strongly influenced by social and economic factors and the functioning of the healthcare systems (9). Weak healthcare systems often amplify disaster health impacts. Figure 2 illustrates the complex pathways of how human health and health systems are affected and the interrelation of these factors in the aftermath of disasters.

The time to preparedness varies by disaster type, influences response strategies, and is further compounded by the generalized, largely reactive approaches to disaster management at all levels, particularly in the low- and middle-income countries (10). The ‘Disaster Characteristics Assessment Scale’ helps locate various disasters on a scale of predictability, lethality, scope, and onset delay and is useful in grading disasters and guiding preparedness policies (2, 11).

A significant pool of research exists on the health and health systems impacts on the various disasters. However, in general, disaster data are incomplete and lacks standardization in terms of collecting, processing, and reporting (12). Furthermore, definitions and time frames used differ affecting quality (10). In terms of health impacts, injury epidemiology is well documented, particularly after earthquakes but comparability is weak. Infectious diseases data and mental health assessments are mostly conducted in high-income countries and evidence from low- and middle-income countries is rare (9).

Another aspect of studying health and health systems impacts of natural disasters are the approaches used. What is essentially overlooked in traditional health impact assessments is that the resilience of a community to combat an impact is a function of the sociodemographic, socioeconomic, sociopolitical, sociopsychological, sociocultural, and sociostructural variables (13). Furthermore, the total economic impact of a disaster constitutes the valuation of its impact on the public goods (hospitals, infrastructure, etc.), private goods (houses, agricultural production, etc.), public health (mortality, morbidity, disability, etc.), and the environment (soil, water quality, etc.) (14). A complete assessment of economic damage is essential although rarely achievable, particularly in low- and middle-income countries. In addition, in general, there is a dearth of original economic valuation studies from low- and middle-income countries to inform policies (14). Thus, the close inter-relations between social, health, and economic impacts significantly substantiate setbacks to overall development. Nonetheless, the focus still remains largely on isolated impact assessments. Developing and standardizing assessment tools and innovative applications of existing tools are necessary to improve the understanding of the complete disaster picture but are generally lacking.

## The way forward

Hoping to go beyond the theoretical boundaries that lead to more simplistic and descriptive dichotomies of health and healthcare impacts, there is a need to understand associated impacts of natural disasters on human life that include the social and the economic sectors. There is an urgency to further our understanding of cumulative integrated impacts and effectively develop evidence-based preparedness plans. We believe that holistic mitigation and preparedness will go a long way in strengthening coping mechanisms at both micro and macro levels to deal with future disasters.

The goal of the MICRODIS project was to investigate the MICRO level health, social and economic impacts of DISasters on communities in Asia and Europe and to generate evidence to inform preparedness, mitigation, and prevention strategies. The focus was first to understand the challenges in measuring integrated impacts and second to develop appropriate methodological tools that can adequately assess them. Integrated impact assessment tools were developed and field tested at 10 sites across Asia and Europe.

Preliminary findings were presented at a scientific symposium held in November 2010 at the University of Heidelberg. Out of these findings and presentations, nine selected articles were submitted to *Global Health Action* and compiled in a special volume on the impact of natural disasters on health, social, and economic systems.

### Overview of the special volume

The nine articles in this special volume focus on describing the intricacies and interdependencies of health, social, and economic impacts of natural disasters on human life, although the main focus converges onto the health and health systems impacts for a majority of them.

1. The introductory article by Sauerborn et al. gives an overview on the current debate on causalities between climate change and disasters. In particular, the authors discuss the following questions: Have some types of extreme events become more frequent, severe, and longer in the past decades? What is the relative contribution of climate change to various types of extreme events? How can the researchers from health, climate and development studies coordinate and cooperate to protect health impacts of natural disasters? (15).
2. Wind et al. explored the association between social capital and disaster mental health in a community-based, cross-sectional study after the flood in Morpeth, Northern England (16). They put forth an interesting proposition stating that affected people may benefit from a combination of individual stress reducing interventions and psychosocial interventions that foster cognitive social capital. How these findings can be translated into intervention programs constitute the subsequent steps. Building on social capital to innovatively combat mental health impacts is an essential point, especially given that mental health is often neglected in the initial phases of disasters.
3. Injury forms a significant disaster health burden. Phalkey et al. investigated the epidemiology of injuries in the first 10 weeks after an earthquake in Gujarat, India, and observed similar patterns across earthquakes but point to the non-standardization of injury data recording, which severely limits generalizability of findings from individual studies. The evidence base for policy planning is limited and building quality databases for recording health impacts is mandated (17). Mis-management or inadequate management of injuries often lead to disabilities and are rarely documented in any follow-up studies (18, 19).
4. Sundaryo et al. seconded this through their study after the Padang earthquake in Indonesia and argue that through disability, injury may contribute to decreased quality of life. Physical injury is significantly correlated with both higher disability and lower quality of life, and disability has significant negative correlation with quality of life in their observations (20). Individuals with physical disabilities are 5.6 times more vulnerable to earthquake impacts (21). It is only pragmatic to acknowledge that minimum standards of medical care should be widely advocated and implemented following all disasters, particularly given that these disabilities create future vulnerabilities (22).
5. Reinhardt et al. furthered the post-injury disability discussion in their article, ‘Disability and health-related rehabilitation in international disaster relief’ and stress on the lack of systemic assessment and measurement of disability after natural disasters. They emphasize that health-related rehabilitation potentially reduces the burden of disabling injuries and should figure as an essential component of medical preparedness and response teams (23). Local healthcare systems, which are the first responders, are often not designed to trigger health-related rehabilitation in the immediate aftermath and skilled personnel are essentially lacking. All three articles suggest that injury epidemiology across disasters is patchy and inconclusive in guiding effective response strategies. In view of the fact that physical injuries and disaster disabilities may additionally contribute to mental health and affect livelihoods and hence economics of the disaster impacts at an individual level, developing standardized outcome measures is mandated.
6. Joshi et al. and Bich et al. addressed the health impacts following floods. Bich et al. outlined the spectrum of health impacts following the floods in Hanoi, Viet Nam, and brought out three essential aspects of disasters: the case of dealing with natural disasters in urban settings, inclusion of non-communicable diseases in the immediate aftermath of a flood, and psychological issues that need attention (24). The authors highlight the need for action on outlined prevention strategies, which seem to be taken for granted in areas with recurrent flooding.
7. Children form a significant proportion of the affected population after natural disasters and pose special challenges in terms of treatment and healthcare. Joshi et al. addressed the case of diarroheal diseases in the flood-prone Bahraich district of Uttar Pradesh in India (25). The article outlines the differences in manifestations of immediate and long-term health impacts of floods, effects of premorbid condition, and economic status of the family on health outcomes of children. They also investigate the cumulative impacts of recurrent flooding on the prevalence of diarrhoea and suggest variable associations between socioeconomic and demographic factors indicating that integrated impact assessments are the way forward in understanding the true picture of disaster impacts in a community.
8. Phalkey et al. drew attention to the importance of preparedness of healthcare systems in areas of recurrent flooding (26). They investigated the functional capacity of the public healthcare system in the Jagatsinghpur district of rural Orissa, India, to face annual floods and reported that individual facility preparedness plans should be developed as an adjunct to local plans. They argue that simple standard operating procedures and distributed stockpiling go a long distance in limiting health impacts after disasters. There is an urgency to convert ‘culture of disasters’ to ‘culture of preparedness’ and building resilient healthcare systems is the essential first step in the direction.
9. Economics of disasters are complex and are intricately interwoven with the social and health conditions of the affected. Evaluations are conducted both ex ante to assess cost benefits of preparedness, preventive interventions, and mitigation to reduce losses from disasters and ex post to assess compensatory assistance to help recover from the impacts. Navrud et al. pioneered an innovative approach of asking about willingness to contribute (WTC) in kind through labor to a flood prevention program as a measure of welfare loss experienced by households due to flooding in Quang Nam province of Central Viet Nam (27). They successfully evaded the main problems associated with applying contingent valuation (CV) approaches in developing countries and concluded that using WTC labor instead of money is promising to capture total welfare loss from natural disasters and should be applied to other disaster settings.

This provides evidence that the application of innovative methodological approaches can go a long way in understanding disaster impacts in a holistic way.

*Michael Marx* and *Revati Phalkey* Institute of Public Health, Medical School University of Heidelberg, Heidelberg, Germany michael.marx@urz.uni-heidelberg.de*Debarati Guha-Sapir* Centre for Research on the Epidemiology of Disasters (CRED) Université Catholique de Louvain, School of Public Health, Brussels, Belgium
